# A Daily Need

**Published:** 1875-10

**Authors:** 


### A DAILY NEED.

That very useful institution, The Packer Manufacturing Company, makes a fine display of their goods, at the Exhibition of the American Institute. Their business is the manufacture of soaps, and no topic upon which we can write is fraught with greater importance to the human race than this same soap-topic.

If there is a single need which cleanly humanity must admit, it is the need of appropriate means for *keeping* cleanly. We must have water in abundance, or we are miserable; we must have clean towels, or life is a burden; we must have soap, or the touch of all things which soil and defile, clings to us, and makes us sad. Happily, the average American lacks not water; and, having this, if he lacks cleanliness, he alone is to blame. What shall we say for his soap?

Well, we are not permitted to say much, in a commendatory vein. Most soaps used upon the skin are bad, vicious, obnoxious. They are made of bad substances, unwholesome substances, abominable substances. They cleanse the skin because they contain free alkali, which dissolves the dirt and a little of the skin, too. But they are rarely a bland, emollient, comforting thing,—soothing while cleansing, allaying irritation while making the cuticle pure and sweet and wholesome. All this a true soap for human contact ought to be and do.

Hundreds of thousands of families keep a jar into which all sorts of refuse grease is emptied. By and by the soap-fat man comes along, and carries away this unsavory mess. It comes back in a thousand forms, under beautiful names, its pollution hidden under sweet floral epithets.

A poor over-worked horse dies on the highway, and his body is taken possession of by the same maker of soap. It matters not if the carcass has gone forward in the process of decomposition until the very air is poisoned for blocks around by the unwholesome stench; the material for soap is there, and the festering mass is destined to be rubbed upon human hands and faces and limbs. This is horrible!

How many diseases of the skin do physicians find which do not yield to treatment, and will not, because kept alive by the original cause of polluted innoculation—diseased soap.

It is not only possible to have good wholesome soap, but it is even possible to have soap which shall contain within itself elements healing, soothing and refreshing. It is possible to medicate soap, so as to confer upon it absolute remedial powers. The era of unclean ointments has passed. Few doctors now direct patients to lumber and clog the skin with greasy unguents; soap becomes the vehicle for conveying the medicine.

We have used more soaps than we can name, but we have never used any soap with the satisfaction and blessed results which have attended the employment of Packer's Pine-Tar Soap. We tried it first, because a good friend and physician advised us to. We admired it, and now never lose a chance to say that it is the most comforting and soothing soap we ever saw. We use it for shaving and for washing, and carry a cake of it around for use upon ulcers of every class, upon all offensive discharges, and in all cases of exzema and herpes. It works to perfection in such cases, and is sufficiently potent to improve the appearance of a majority of old sores, and to rapidly facilitate the healing process. As a deodorizer it is wonderful,—destroying the odors of perspiration on the feet, or any portion of the body. In short, it is a perfect soap, while being at the same time vastly more than soaps in general, viz: a splendid adjunct to the healing art.

